# Herd Immunity against Severe Acute Respiratory Syndrome Coronavirus 2 Infection in 10 Communities, Qatar

**DOI:** 10.3201/eid2705.204365

**Published:** 2021-05

**Authors:** Andrew Jeremijenko, Hiam Chemaitelly, Houssein H. Ayoub, Moza Alishaq, Abdul-Badi Abou-Samra, Jameela Ali A.A. Al Ajmi, Nasser Ali Asad Al Ansari, Zaina Al Kanaani, Abdullatif Al Khal, Einas Al Kuwari, Ahmed Al-Mohammed, Naema Hassan Abdulla Al Molawi, Huda Mohamad Al Naomi, Adeel A. Butt, Peter Coyle, Reham Awni El Kahlout, Imtiaz Gillani, Anvar Hassan Kaleeckal, Naseer Ahmad Masoodi, Anil George Thomas, Hanaa Nafady-Hego, Ali Nizar Latif, Riyazuddin Mohammad Shaik, Nourah B.M. Younes, Hanan F. Abdul Rahim, Hadi M. Yassine, Mohamed G. Al Kuwari, Hamad Eid Al Romaihi, Mohamed H. Al-Thani, Roberto Bertollini, Laith J. Abu-Raddad

**Affiliations:** Hamad Medical Corporation, Doha, Qatar (A. Jeremijenko, M. Alishaq, A.-B. Abou-Samra, J.A.A.A. Al Ajmi, N.A.A. Al Ansari, Z. Al Kanaani, A. Al Khal, E. Al Kuwari, A. Al-Mohammed, N.H.A. Al Molawi, H.M. Al Naomi, A.A. Butt, P. Coyle, R.A. El Kahlout, I. Gillani, A.H. Kaleeckal, N.A. Masoodi, A.G. Thomas, H. Nafady-Hego, A.N. Latif, R.H. Shaik, N.B.M. Younes);; Weill Cornell Medicine–Qatar of Cornell University, Doha (H. Chemaitelly, L.J. Abu-Raddad);; World Health Organization Collaborating Centre for Disease Epidemiology Analytics on HIV/AIDS, Sexually Transmitted Infections, Doha (H. Chemaitelly, L.J. Abu-Raddad);; Qatar University, Doha (H.H. Ayoub, H.F. Abdul Rahim, H.M. Yassine);; Assiut University, Assiut, Egypt (H. Nafady-Hego);; Primary Health Care Corporation, Doha (M.G. Al Kuwari);; Ministry of Public Health, Doha (H.E. Al Romaihi, M.H. Al-Thani, R. Bertollini);; Cornell University, New York, New York, USA (A.A. Butt, L.J. Abu-Raddad)

**Keywords:** severe acute respiratory syndrome coronavirus 2, SARS-CoV-2, coronavirus, viruses, coronavirus disease, COVID-19, epidemiology, infection, respiratory infections, seroprevalence, communities, craft and manual workers, CMWs, herd immunity, immunity, zoonoses, Qatar

## Abstract

We investigated what proportion of the population acquired severe acute respiratory syndrome coronavirus 2 (SARS-CoV-2) infection and whether the herd immunity threshold has been reached in 10 communities in Qatar. The study included 4,970 participants during June 21–September 9, 2020. Antibodies against SARS-CoV-2 were detected by using an electrochemiluminescence immunoassay. Seropositivity ranged from 54.9% (95% CI 50.2%–59.4%) to 83.8% (95% CI 79.1%–87.7%) across communities and showed a pooled mean of 66.1% (95% CI 61.5%–70.6%). A range of other epidemiologic measures indicated that active infection is rare, with limited if any sustainable infection transmission for clusters to occur. Only 5 infections were ever severe and 1 was critical in these young communities; infection severity rate of 0.2% (95% CI 0.1%–0.4%). Specific communities in Qatar have or nearly reached herd immunity for SARS-CoV-2 infection: 65%–70% of the population has been infected.

Since the start of the severe acute respiratory syndrome coronavirus 2 (SARS-CoV-2) pandemic, millions of infections have been confirmed through real-time reverse transcription PCR (RT-PCR) testing (*1*), and millions probably have gone undocumented (*2*). Two key questions remain unanswered. Has any community reached herd immunity to render infection transmission chains unsustainable? What proportion of the population needs to be infected to reach herd immunity?

Qatar, a peninsula in the Arabian Gulf region that has a diverse population of 2.8 million (*3*), has experienced a large-scale SARS-CoV-2 epidemic (*4**,**5*). By January 14, 2021, the rate of real-time RT-PCR–confirmed infections exceeded 65 cases/1,000 persons (*6*). The epidemic, which is currently in an advanced stage (*4*), seems to have followed a classic susceptible-infected-recovered pattern, with an epidemic peak around May 20, followed by a steady decrease for the next 8 months (*4*).

The subpopulation most affected by this epidemic was expatriate craft and manual workers (CMWs) among whom community transmission was first identified (*4*). These workers constitute ≈60% of the population in Qatar and are typically single men 20–49 years of age (*7*). CMWs at a given workplace or company not only work together during the day but also live together as a community in large dormitories or housing complexes in which they share rooms, bathrooms, and cafeteria-style meals (*4**,**8*). These communities stay mostly in contact with their own community members and infrequently mingle with other communities, creating a geographic bubble that proved essential for the pattern of infection transmission (*4*). With reduced options for effective social and physical distancing, SARS-CoV-2 transmission in these CMW communities resembled that of influenza outbreaks in schools (*4**,**9**,**10*), and especially boarding schools (*10*). This finding is observed despite implementation of nonpharmaceutical control measures, such as a mask mandate after the World Health Organization (WHO) recommendation (*11*), promotion and facilitation of social and physical distancing, disinfection of surfaces, and awareness messaging in different languages. A similar transmission pattern has been documented among migrant workers in Singapore (*12**,**13*) and Spain (*14*).

Factors observed included the large number of diagnosed infections in CMWs (*4*), the large proportion of infections that were asymptomatic (*4*,*15*,*16*), the high real-time RT-PCR positivity rates in the random testing campaigns conducted around the epidemic peak in different CMW communities (*4*), the observed susceptible-infected-recovered epidemic curve with steady decreases in incidence for 8 months despite the gradual easing of the social and physical distancing restrictions (*4*,*17*), and evidence indicating an efficacy >90% for natural infection against reinfection that lasts for >7 months (*18*; L.J. Abu-Raddad et al., unpub. data). All of these factors raised questions of whether herd immunity might have been reached in at least some of these communities.

On the basis of these considerations, we hypothesized that at least some of the CMW communities have already reached the herd immunity threshold. To investigate this hypothesis, our specific objective was to assess the proportion of the population that has been infected by assessing the level of detectable antibodies. More than 90% of real-time RT-PCR–confirmed cases in Qatar show development of detectable antibodies (*4*); therefore, we operationally defined herd immunity as the proportion of the population that needs to have detectable antibodies before infection transmission/circulation becomes unsustainable in this population, with limited if any new infections occurring. The study was conducted to inform the national response and preparedness for potential future infection waves.

## Methods

### Data Sources

We conducted testing for detectable SARS-CoV-2–specific antibodies in blood specimens in 10 CMW communities during June 21–September 9, 2020. This testing was part of an a priori–designed study combined with a testing and surveillance program led by the Ministry of Public Health and Hamad Medical Corporation (HMC), the main public healthcare provider in Qatar and the nationally designated provider for all COVID-19 healthcare needs. The goal of this program was to assess the level of infection exposure in different subpopulations and economic sectors.

The study design was opportunistic using the Ministry of Public Health–HMC program and the need for rapid data collection to inform the national response. We specifically selected the 10 CMW communities for feasibility or given earlier random real-time RT-PCR testing campaigns or contact tracing that suggested substantial infection levels. For instance, CMW community 1 was part of a random real-time RT-PCR testing campaign that identified, by using nasopharyngeal swab specimens, a high positivity rate of 59% during late April 2020.

The population size of each of these communities ranged from a few hundred to a few thousand who live in shared accommodations provided by the employers. The companies that employ these workers belonged to the service or industrial sectors, but the bulk of the employees, even in the industrial companies, worked on providing services, such as catering, cleaning, and other janitorial services, warehousing, security, and port work.

Ten employers were contacted and were willing to participate and advertise the availability and location of testing sites to their employees. Participation was voluntary. Employees interested in being tested and in knowing their status were provided with transportation to HMC testing sites. Informed consent was able to be obtained in 9 languages (Arabic, Bengali, English, Hindi, Urdu, Nepali, Sinhala, Tagalog, and Tamil) to cater to the main language groups spoken in the CMW communities of Qatar.

We used self-administered questionnaires in these same languages only for CMW community 1; questionnaires were given by trained public health workers to collect data on sociodemographics and history of exposure and symptoms. We developed the questionnaire on the basis of suggestions from WHO (*19*). A blood specimen was obtained from all study participants, and in 6 communities, nasopharyngeal swab specimens were simultaneously collected for real-time RT-PCR testing by licensed nurses. We applied national guidelines and standard of care to all identified real-time RT-PCR–positive case-patients, including requirement of isolation and other measures to prevent infection transmission. No action was mandated by the national guidelines to those persons found to be antibody positive but real-time RT-PCR negative, and thus no action was taken apart from notifying persons of their serostatus.

We subsequently linked results of the serologic testing to the HMC centralized and standardized database comprising all SARS-CoV-2 real-time RT-PCR testing conducted in Qatar since the start of the epidemic (*4*). The database also includes data on hospitalization and on the WHO severity classification (*20*) for each real-time RT-PCR–confirmed infection. Data were also linked to datasets of 2 recently completed national reinfection studies (*18*; L.J. Abu-Raddad et al., unpub. data) to identify reinfections. The study was approved by HMC and Weill Cornell Medicine–Qatar Institutional Review Boards.

### Laboratory Methods

We performed testing for SARS-CoV-2–specific antibodies in serologic samples by using an electrochemiluminescence immunoassay (Roche Elecsys Anti-SARS-CoV-2, https://www.roche.com) (sensitivity 99.5%, specificity 99.8%) (*21*,*22*). We interpreted results according to the manufacturer’s instructions: reactive for a cutoff index ≥1.0 and nonreactive for a cutoff index <1.0 (*22*).

We performed real-time RT-PCR testing of aliquots of universal transport medium (Huachenyang Technology, https://szhcy.en.alibaba.com) used for collection of nasopharyngeal swab specimens. We extracted aliquots by using the QIAsymphony Platform (QIAGEN, https://www.qiagen.com); tested them by using real-time PCR (TaqPath COVID-19 Combo Kit; Thermo Fisher Scientific, https://www.thermofisher.com (sensitivity 100%, specificity 100%) (*23*) in an ABI 7500 FAST System (ThermoFisher); extracted them by using a custom protocol (M.K. Kalikiri et al., Sidra Medicine, pers. comm., 2021 Feb 1) on a Hamilton Microlab STAR (https://www.hamiltoncompany.com); tested them by using the AccuPower SARS-CoV-2 Real-Time RT-PCR Kit (Bioneer, https://www.bioneer.com) (sensitivity 100%, specificity 100%)   (*24*) on an ABI 7500 FAST System or loaded them directly into a Roche Cobas 6800 system; and assayed them by using the Cobas SARS-CoV-2 Test (sensitivity 95%, specificity 100%) (*25*). All laboratory testing was conducted at HMC Central Laboratory following standardized protocols.

### Statistical Analysis

We used frequency distributions to describe characteristics of CMWs and to estimate different SARS-CoV-2 epidemiologic measures. We estimated the pooled mean for SARS-CoV-2 seropositivity across CMW communities by using meta-analysis. We applied a DerSimonian-Laird random-effects model (*26*) to pool seroprevalence measures that were weighted by using the inverse-variance method (*27**,**28*).

We used χ^2^ tests and univariable logistic regressions to determine the association of each prespecified covariate (i.e., sex, age, nationality, and CMW community) with seropositivity. For CMW community 1, we also investigated associations of educational attainment, contact with an infected person, presence of symptoms in the previous 2 weeks, and whether symptoms required medical attention with seropositivity. Missing values were included as separate subcategories in the analyses. We generated summary statistics, as well as odds ratios (ORs), 95% CIs, and p values (Tables 1, 2; Appendix Tables 1, 2).

**Table 1:** Characteristics of 10 CMWs and associations with SARS-CoV-2 seropositivity, indicated by detectable antibodies in serologic samples, Qatar*

Characteristic	No. (%)† tested	SARS-CoV-2 seropositive				Univariable regression analysis			Multivariable regression analysis‡	
		No.	%§ (95% CI)	p value		OR (95% CI)	p value¶		OR (95% CI)	p value#
Sex										
M	4,721 (95.0)	3,153	66.8 (65.4–68.1)	<0.001		Referent			Referent	
F	249 (5.0)	46	18.5 (13.9–23.9)			0.11 (0.08–0.16)	<0.001		0.13 (0.09–0.19)	<0.001
Age, y										
<29	1,579 (31.8)	1,031	65.3 (62.9–67.6)	<0.001		Referent			Referent	
30–39	1,973 (39.7)	1,226	62.1 (60.0–64.3)			0.87 (0.76–1.00)	0.052		0.90 (0.78–1.05)	0.178
40–49	1,040 (20.9)	680	65.4 (62.4–68.3)			1.00 (0.85–1.18)	0.962		1.12 (0.93–1.35)	0.216
>50	339 (6.8)	225	66.4 (61.1–71.4)			1.05 (0.82–1.34)	0.705		1.21 (0.92–1.59)	0.170
Missing	39 (0.8)	37	94.9 (82.7–99.4)			9.83 (2.36–40.95)	0.002		9.57 (2.22–41.32)	0.002
Nationality										
Other**	125 (2.5)	40	32.0 (23.9–40.9)	<0.001		Referent			Referent	
Filipino	186 (3.7)	68	36.6 (29.6–43.9)			1.22 (0.76–1.98)	0.408		2.23 (1.32–3.75)	0.003
Sri Lankan	147 (3.0)	77	52.4 (44.0–60.7)			2.34 (1.42–3.84)	0.001		2.81 (1.66–4.76)	<0.001
Kenyan	152 (3.1)	77	50.7 (42.4–58.9)			2.18 (1.33–3.57)	0.002		3.43 (1.99–5.90)	<0.001
Indian	1,647 (33.1)	1,035	62.8 (60.5–65.2)			3.59 (2.44–5.30)	<0.001		3.60 (2.40–5.41)	<0.001
Nepalese	2,136 (43.0)	1,468	68.7 (66.7–70.7)			4.67 (3.17–6.88)	<0.001		4.93 (3.27–7.42)	<0.001
Bangladeshi	577 (11.6)	434	75.2 (71.5–78.7)			6.45 (4.23–9.82)	<0.001		6.78 (4.31–10.66)	<0.001
CMW community										
5	443 (8.9)	243	54.9 (50.1–59.6)	<0.001		Referent			Referent	
4	534 (10.7)	330	61.8 (57.5–65.9)			1.33 (1.03–1.72)	0.028		1.12 (0.83–1.52)	0.449
10	957 (19.3)	620	64.8 (61.7–67.8)			1.51 (1.20–1.90)	<0.001		1.30 (1.02–1.65)	0.034
7	188 (3.8)	122	64.9 (57.6–71.7)			1.52 (1.07–2.17)	0.020		1.31 (0.91–1.89)	0.154
6	1,505 (30.3)	946	62.9 (60.4–65.3)			1.39 (1.12–1.73)	0.002		1.32 (1.06–1.66)	0.015
2	456 (9.2)	282	61.8 (57.2–66.3)			1.33 (1.02–1.74)	0.034		1.46 (1.08–1.96)	0.013
9	202 (4.1)	126	62.4 (55.3–69.1)			1.36 (0.97–1.92)	0.074		1.71 (1.18–2.48)	0.005
8	139 (2.8)	93	66.9 (58.4–74.6)			1.66 (1.12–2.48)	0.013		1.92 (1.25–2.95)	0.003
1	255 (5.1)	193	75.7 (69.9–80.8)			2.56 (1.82–3.61)	<0.001		2.52 (1.75–3.62)	<0.001
3	291 (5.9)	244	83.8 (79.1–87.9)			4.27 (2.97–6.15)	<0.001		3.49 (2.41–5.07)	<0.001

*CMW, craft and manual worker; OR, odds ratio; SARS-CoV-2, severe acute respiratory syndrome coronavirus 2. †Percentage of total sample. ‡Pseudo-R^2^ value in the multivariable logistic regression model is 7.1%. §Percent seropositive of those tested. ¶Covariates with p <0.2 in univariable analysis (i.e., sex, age, nationality, and CMW community) were included in the multivariable analysis. #Covariates with p <0.05 in multivariable analysis (i.e., sex, nationality, and CMW community) were considered predictors of SARS-CoV-2 seropositivity. **Includes all other nationalities that contributed <10% of the sample in each community. These are: Canadian, Egyptian, Ethiopian, Georgian, Ghanaian, Indonesian, Iraqi, Jordanian, Lebanese, Nigerian, Pakistani, Palestinian, Somali, Tanzanian, Tunisian, Ugandan, and Yemeni.

**Table 2:** Characteristics of CMW community 1 and associations with SARS-CoV-2 seropositivity (detectable antibodies in serologic samples) including sociodemographics, history of exposure, and symptoms, Qatar*

Characteristic	No. (%)† tested	SARS-CoV-2 seropositive				Univariable regression analysis‡	
		No.	%§ (95% CI)	p value		OR (95% CI)	p value¶
Sex							
M	240 (94.1)	189	78.8 (73.0–83.7)	<0.001		Referent	
F	15 (5.9)	4	26.7 (7.8–55.1)			0.10 (0.03–0.32)	<0.001
Age, y							
<29	105 (41.2)	84	80.0 (71.1–87.2)	0.322		Referent	
30–39	111 (43.5)	83	74.8 (65.6–82.5)			0.74 (0.39–1.41)	0.360
40–49	27 (10.6)	19	70.4 (49.8–86.2)			0.59 (0.23–1.54)	0.284
>50	12 (4.7)	7	58.3 (27.7–84.8)			0.35 (0.10–1.21)	0.098
Nationality							
Other#	48 (18.8)	23	47.9 (33.3–62.8)	<0.001		Referent	
Indian	32 (12.5)	20	62.5 (43.7–78.9)			1.81 (0.73–4.51)	0.202
Nepalese	157 (61.6)	132	84.1 (77.4–89.4)			5.74 (2.82–11.67)	<0.001
Bangladeshi	18 (7.1)	18	100.0 (81.5–100.0)			Omitted by model	NA
Education level							
Intermediate or lower	101 (39.6)	88	87.1 (79.0–93.0)	<0.001		Referent	
Secondary/high school/vocational	80 (31.4)	69	86.3 (76.7–92.9)			0.93 (0.39–2.20)	0.863
University	27 (10.6)	17	63.0 (42.4–80.6)			0.25 (0.09–0.67)	0.005
Missing	47 (18.4)	19	40.4 (26.4–55.7)			0.10 (0.04–0.23)	<0.001
Contact with an infected person							
No	124 (48.6)	93	75.0 (66.4–82.3)	0.303		Referent	
Yes	14 (5.5)	13	92.9 (66.1–99.8)			4.33 (0.54–34.48)	0.166
Unknown/missing	117 (45.9)	87	74.4 (65.5–82.0)			0.97 (0.54–1.73)	0.909
Symptoms in the past 2 weeks**							
Asymptomatic	184 (72.2)	148	80.4 (74.0–85.9)	<0.001		Referent	
1	16 (6.3)	16	100.0 (79.4–100.0)			Omitted by model	NA
>2	12 (4.7)	12	100.0 (73.5–100.0)			Omitted by model	NA
Missing	43 (16.9)	17	39.5 (25.0–55.6)			0.16 (0.08–0.32)	<0.001
Symptoms required medical attention							
No	210 (82.4)	174	82.9 (77.1–87.7)	<0.001		Referent	
Yes	3 (1.2)	3	100.0 (29.2–100.0)			Omitted by model	NA
Unknown/missing	42 (16.5)	16	38.1 (23.6–54.4)			0.13 (0.06–0.26)	<0.001

*CMW, craft and manual worker; NA, not applicable; OR, odds ratio; SARS-CoV-2, severe acute respiratory syndrome coronavirus 2. †Percentage of total sample. ‡Overall sample size and numbers per stratum were too small to warrant conduct of meaningful multivariable regression analysis. §Percent seropositive of those tested. ¶Covariates with p <0.05 in univariable analysis (i.e., sex, nationality, and education level were considered as showing evidence for an association with SARS-CoV-2 seropositivity. #Includes all other nationalities that contributed <10% of the sample. These are Filipino, Georgian, Kenyan, Sri Lankan, and Tunisian. **Symptoms were based on self-report and included fever, chills, muscle ache/myalgia, sore throat, cough, runny nose/rhinorrea, shortness of breath, wheezing, chest pain, other respiratory symptoms, headache, nausea and vomiting, abdominal pain, diarrhea, loss of sense of smell, and loss of sense of taste.

We performed multivariable logistic regressions to estimate the magnitude of the association of a specific covariate adjusting for other covariates in the model. Covariates with p values <0.2 in univariable regression analysis were included simultaneously in the multivariable logistic regression model. Covariates with p values <0.05 in the multivariable model were considered as showing evidence for an association with the outcome, and associated adjusted ORs (aORs), 95% CIs, and p values were generated and reported (Tables 1, 2; Appendix Tables 1, 2). No interactions were investigated. Statistical models’ goodness of fit were reported. The distribution of real-time RT-PCR cycle threshold (C_t_) values for persons who were real-time RT-PCR positive was further generated, and summary statistics were reported. Statistical analyses were performed by using STATA/SE version 16.1 (https://www.stata.com) (*29*).

We also conducted mathematical modeling simulations to highlight the effect of heterogeneity in the risk for exposure to the infection on the level of herd immunity. These simulations were generated by using a classic age-structured, susceptible-exposed-infectious-recovered mathematical model published elsewhere (*17*). Simulations were implemented by using MATLAB R2019a (https://www.mathworks.com) (*30*).

## Results

A total of 4,970 CMWs from the 10 CMW communities participated in this study (Table 1). Participants were mostly men (95.0%); <40 years of age (71.5%); and of Nepalese (43.0%), Indian (33.1%), or Bangladeshi (11.6%) origin. Regression analyses identified each of sex, nationality, and CMW community to be independently associated with seropositivity.

Women had 87% lower odds of being seropositive than men (aOR 0.13, 95% CI 0.09–0.19) (Table 1). Compared with all other nationalities (Table 1), aOR was 6.78 (95% CI 4.31–10.66) for Bangladeshis, 4.93 (95% CI 3.27–7.42) for Nepalese, 3.60 (95% CI 2.40–5.41) for Indians, 3.43 (95% CI 1.99–5.90) for Kenyans, 2.81 (95% CI 1.66–4.76) for Sri Lankans, and 2.23 (95% CI 1.32–3.75) for Filipinos. Some differences in seropositivity by CMW community were noted (Table 1). No major differences in seropositivity by age group were found (Table 1).

We provide characteristics and associations with seropositivity (detectable antibodies in serologic samples) for only CMW community 1, in which a specific self-administered questionnaire was administered and collected specific sociodemographic data and history of exposure and symptoms (Table 2). Nearly 40% of participants had intermediate or low educational attainment, and 31% had higher schooling levels or vocational training. University education was associated with a 75% (OR 0.25, 95% CI 0.09–0.67) lower odds of seropositivity compared with intermediate or lower educational attainment. No significant associations with seropositivity were found for contact with an infected person, presence of symptoms, or symptoms requiring medical attention. We provide characteristics and associations with SARS-CoV-2 seropositivity for CMW communities 2–10 (Appendix Table 1). For each of these communities, associations were found for sex and nationality, but no major associations were found for age group.

**Figure 1:**
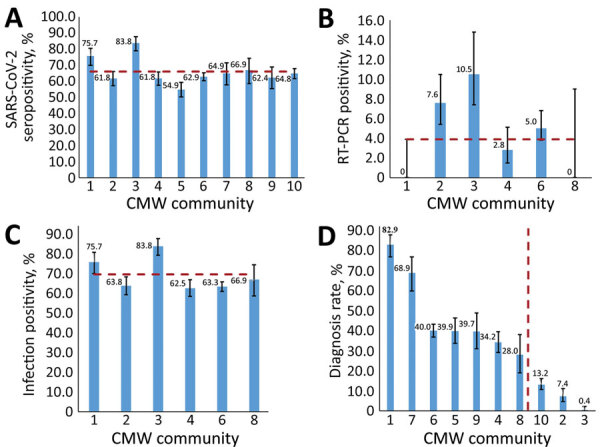
Measures of SARS-CoV-2 infection across 10 craft and manual worker communities, Qatar. A) Seropositivity (antibody positivity), B) real-time RT-PCR positivity, C) infection positivity (antibody or real-time RT-PCR positive), and D) diagnosis rate. Panels B and D show results for only the 6 communities for whom real-time RT-PCR testing was performed. Percentages are shown above bars. Numbers along the x-axes of each panel indicate the community number. Error bars indicate 95% CIs. CMW, craft and manual workers; RT-PCR, real-time reverse transcription PCR; SARS-CoV-2, severe acute respiratory syndrome coronavirus 2.

We provide key SARS-CoV-2 epidemiologic measures in the different CMW communities (Figure 1). Of 4,970 SARS-CoV-2 antibody test results for these CMWs, 3,199 (64.4%, 95% CI 63.0%–65.7%) were seropositive. Seropositivity ranged from 54.9% (95% CI 50.2%–59.4%) for CMW community 5 to 83.8% (95% CI 79.1%–87.7%) for CMW community 3 (Figure 1, panel A). The pooled mean for SARS-CoV-2 seropositivity across the 10 CMW communities was 66.1% (95% CI 61.5%–70.6%).

**Figure 2:**
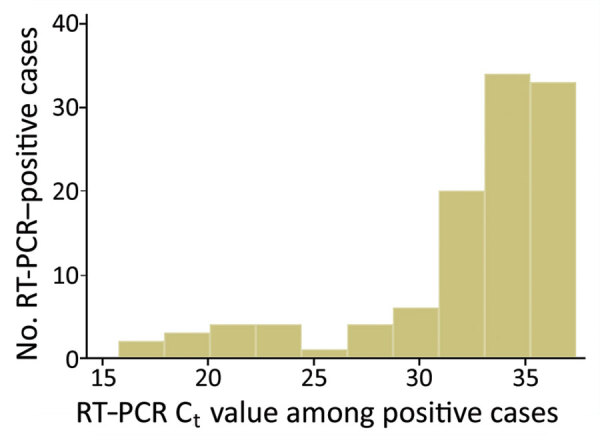
Distribution of real-time RT-PCR C_t_ values among craft and manual workers identified as real-time RT-PCR positive for severe acute respiratory syndrome coronavirus 2, Qatar. C_t_, cycle threshold; RT-PCR, real-time reverse transcription PCR.

Of 2,016 real-time RT-PCR tests using nasopharyngeal swab specimens collected during this study for these CMWs, 112 (5.6%, 95% CI 4.6%–6.6%) were positive. Real-time RT-PCR positivity ranged from 0.0% (95% CI 0.0%–3.9%) for CMW community 1 and 0.0% (95% CI 0.0%–9.0%) for CMW community 8 to 10.5% (95% CI 7.4%–14.8%) for CMW community 3 (Figure 1, panel B). Pooled mean real-time RT-PCR positivity across the 6 CMW communities in which real-time RT-PCR testing was conducted was 3.9% (95% CI 1.6%–6.9%). The C_t_ values ranged from 15.8 to 37.4 (median 34.0) (Figure 2). Most (79.5%) real-time RT-PCR–positive persons had C_t_ values >30, suggestive of no active infection (*31**,**32*). Major differences in real-time RT-PCR positivity were found by nationality and CMW community (Appendix Table 2).

Infection positivity (antibody or real-time RT-PCR positive) ranged from 62.5% (95% CI 58.3%–66.7%) for CMW community 4 to 83.8% (95% CI 79.1%–87.7%) for CMW community 3 (Figure 1, panel C). Pooled mean infection positivity across the 6 CMW communities with antibody and RT-PCR results was 69.5% (95% CI 62.8%–75.9%).

Data were linked to the national SARS-CoV-2 real-time RT-PCR testing and hospitalization database. Of the 3,199 antibody-positive CMWs, 1,012 (31.6%, 95% CI 30.0%–33.3%) were previously given a diagnosis of SARS-CoV-2 infection (had a record of a real-time RT-PCR–confirmed positive result before this study). No records of previous real-time RT-PCR positive test results were found for the remaining 2,187 antibody-positive CMWs. For the CMW communities that were previously part of broad real-time RT-PCR testing because of a case identification or a random testing campaign, the diagnosis rate ranged from 28.0% (95% CI 19.1%–38.2%) for CMW community 8 to 82.9% (95% CI 76.8%–87.9%) for CMW community 1. In instances in which no such broad real-time RT-PCR testing was conducted, the diagnosis rate was only 13.2% (95% CI 10.7%–16.1%) for CMW community 10, 7.4% (95% CI 4.7%–11.2%) for CMW community 2, and 0.4% (95% CI 0.0%–2.3%) for CMW community 3. Only a small fraction of antibody-negative persons, 14 of 1,771 (0.8%, 95% CI 0.4%–1.3%), had been previously given a diagnosis of being real-time RT-PCR positive (Appendix Table 3).

Of the total sample, 21 persons had a hospitalization record associated with a SARS-CoV-2 infection diagnosis, of whom, infection severity per WHO classification was mild for 5, moderate for 10, severe for 5, and critical for 1. All 21 persons eventually cleared their infection and were discharged from the hospital. All of these persons were also antibody positive. Accordingly, the proportion of those persons who had a confirmed severe or critical infection of the 3,233 persons who had a laboratory-confirmed infection (antibody or real-time RT-PCR positive result) was 0.2% (95% CI 0.1%–0.4%).

We linked our data to records of 2 recently completed studies. These studies, which assessed reinfection in Qatar in a cohort of >130,000 real-time RT-PCR–confirmed infected persons (*18*) and a cohort of >43,000 antibody-positive persons (L.J. Abu-Raddad et al., unpub. data), identified no reinfections in these study participants whose results were confirmed by using viral genome sequencing.

## Discussion

Our results support that herd immunity has been reached (or at least nearly reached) in these CMW communities, and that the level of herd immunity needed for SARS-CoV-2 infection is a proportion of the population infected of ≈65%–70%. This conclusion has been reached considering the following lines of evidence. First, these CMW communities had comparable seroprevalences of ≈65%–70%. Second, real-time RT-PCR positivity was low and most of those who were real-time RT-PCR positive had a high C_t_ suggestive of an earlier rather than recent infection (*31*,*32*). Third, only a few persons had active infection (C_t_ <25) and no major infection cluster was identified in any of these CMW communities during this study or thereafter (suggestive of isolated infections and unsustainable infection transmission for clusters to occur). Fourth, 2 recent studies from Qatar reported an efficacy >90% for natural infection against reinfection for >7 months after primary infection (*18*; L.J. Abu-Raddad et al., unpub. data), in addition to other evidence on the durability of immunity (*33**–**35*). Fifth, the level of 65%–70% infection exposure is in concordance with that predicted by using the classical formula for herd immunity of 1 – 1/R_0_ (*36*,*37*); R_0_, the basic reproduction number, as 2.5–4.0 (*38*,*39*).

Although large clusters of infection were common in such CMW communities before and around the epidemic peak toward end of May, that time is several weeks before the launch of this study. Thus, no such major cluster has been subsequently identified in such CMW communities in Qatar, despite the progressive easing of the social and physical distancing restrictions since June 15, 2020.

These findings indicate that reaching herd immunity in such largely homogenous communities requires high exposure levels of ≈65%–70%. However, true herd immunity might have been reached even at a lower proportion of the population infected. Mathematical modeling indicates that infection exposure for a novel infection (especially in the first cycle) can considerably overshoot the classical herd immunity level of 1 – 1/R_0_, more so if the social contact rate within this community is homogeneous (Appendix Figure 1). Heterogeneity in the social contact rate can reduce the final proportion of the population that needs to be infected to reach herd immunity (Appendix Figure 1) (*37*; R. Aguas et al., Oxford University, pers. comm., 2021 Feb 1).

The severity rate for SARS-CoV-2 infection was low (0.2%), possibly because of the young age of the CMWs. No COVID-19 deaths were reported in these CMW communities. In communities in which no previous, broad real-time RT-PCR testing was conducted, <15% of the antibody-positive persons had ever been given a diagnosis as being real-time RT-PCR positive before this study. There was a large difference in infection exposure between women and men (Table 1). This difference, with the variable proportion of women across these communities, also explains part of the variation seen in the overall seroprevalence across these communities (Figure 1; Appendix Figure 2). This finding might be attributed to women and men living in different housing accommodations and having different work roles. Women, a small minority in these CMW communities, live in small shared accommodations as opposed to the large ones hosting men.

Differences in results by nationality (Table 1), are explained by nearly all Bangladeshis and Nepalese and most Indians being the workers in these communities, because a proportion of Indians and much of the other nationalities held administrative or managerial positions that had lower social contact rates and possibly lived in different accommodations than most of the workers. No major differences in infection exposure by age were found, although there was a tendency for persons >40 years of age to have lower infection exposure (Appendix Table 1), possibly caused by different occupations within these communities.

Our study’s limitations included that, by design, the study was specifically conducted in select CMW communities, and therefore findings might not be representative nor generalizable to the wider CMW population in Qatar. The small and variable proportion of women in these communities suggests that findings might not also be generalizable to women in these communities. Response rate could not be precisely ascertained given uncertainty around the number of CMWs who were aware of the invitation to participate, but on the basis of employer-reported counts of the size of each community, the response rate was >50%, and participants expressed high interest in knowing their antibody status. The validity of study outcomes is contingent on the sensitivity and specificity of the used assays. However, laboratory methods were based on high-quality commercial platforms, and each diagnostic method was validated in the laboratory before its use. The antibody assay is one of the best available and extensively used and investigated commercial platforms; it has a specificity >99.8% (*22*,*40*,*41*), indicating that false-positive results, or positive results due to cross-reactivity with other common cold coronaviruses, are not likely.

In conclusion, some of the CMW communities in Qatar, who constituted ≈60% of the total population (*7*), have reached or nearly reached herd immunity for SARS-CoV-2 infection. Although achieving herd immunity at a national level is difficult within a few months (*42*), herd immunity could be achieved in specific communities within a few months. In such relatively homogenous communities, reaching herd immunity required infection of 65%–70% of the members of the community. These findings suggest that the SARS-CoV-2 epidemic in a homogenous population is likely to be sustainable until at least two thirds of the population become infected. This finding also suggests that a SARS-CoV-2 vaccine needs at least 65%–70% efficacy at universal coverage for herd immunity to be achieved in a population not exposed to SARS-CoV-2 infection (*43*,*44*; H.H. Ayoub et al., unpub. data). Alternatively, herd immunity might be reached at a vaccination coverage of ≈75% if vaccine efficacy is 95%, similar to that of the recently licensed SARS-CoV-2 vaccines (*45*,*46*).

Appendix Additional information on herd immunity against severe acute respiratory syndrome coronavirus 2 infection in 10 communities, Qatar.
