# Supplementary material for: Herd Immunity against Severe Acute Respiratory Syndrome Coronavirus 2 Infection in 10 Communities, Qatar
Source: Emerg Infect Dis. 2021 May;27(5):1343–52. doi: 10.3201/eid2705.204365 (PMC8084480; doi:10.3201/eid2705.204365)
Supplement: Appendix — Additional information on herd immunity against severe acute respiratory syndrome coronavirus 2 infection in 10 communities, Qatar. [file 20-4365-Techapp-s1.pdf]

Article DOI: https://doi.org/10.3201/eid2705.204365

# Herd Immunity against Severe Acute Respiratory Syndrome Coronavirus 2 Infection in 10 Communities, Qatar

## Appendix

**Appendix Table 1.** Characteristics of CMW communities 2–10 and associations with SARS-CoV-2 seropositivity (detectable antibodies in serologic samples), Qatar*

| | | SARS-CoV-2 seropositive | | | Univariable regression analysis | |
|---|---|---|---|---|---|---|
| Characteristic | No. (%)† tested | No. | %‡ (95% CI) | p value | OR (95% CI) | p value§ |
| CMW community 2 | | | | | | |
| Sex | | | | | | |
| M | 385 (84.4) | 270 | 70.1 (65.3–74.7) | <0.001 | 1.00 | |
| F | 71 (15.6) | 12 | 16.9 (9.0–27.7) | | 0.09 (0.04–0.17) | <0.001 |
| Age, y | | | | | | |
| ≤29 | 301 (66.0) | 180 | 59.8 (54.0–65.4) | 0.005 | 1.00 | |
| 30–39 | 143 (31.4) | 99 | 69.2 (61.0–76.7) | | 1.51 (0.99–2.31) | 0.055 |
| 40–49 | 12 (2.6) | 3 | 25.0 (5.5–57.2) | | 0.22 (0.06–0.84) | 0.027 |
| Nationality | | | | | | |
| Other | 47 (10.3) | 29 | 61.7 (46.4–75.5) | <0.001 | 1.00 | |
| Kenyan | 60 (13.2) | 27 | 45.0 (32.1–58.4) | | 0.51 (0.23–1.11) | 0.088 |
| Nepalese | 296 (64.9) | 181 | 61.1 (55.3–66.7) | | 0.98 (0.52–1.84) | 0.942 |
| Bangladeshi | 53 (11.6) | 45 | 84.9 (72.4–93.3) | | 3.49 (1.34–9.07) | 0.010 |
| CMW community 3 | | | | | | |
| Sex | | | | | | |
| M | 291 (100.0) | 244 | 83.8 (79.1–87.9) | NA | NA | |
| F | NA | NA | NA | | NA | NA |
| Age, y | | | | | | |
| ≤29 | 81 (27.8) | 69 | 85.2 (75.6–92.1) | 0.837 | 1.00 | |
| 30–39 | 109 (37.5) | 89 | 81.7 (73.1–88.4) | | 0.77 (0.35–1.69) | 0.520 |
| 40–49 | 79 (27.1) | 68 | 86.1 (76.5–92.8) | | 1.08 (0.44–2.60) | 0.872 |
| ≥50 | 22 (7.6) | 18 | 81.8 (59.7–94.8) | | 0.78 (0.23–2.72) | 0.700 |
| Nationality | | | | | | |
| Other | 5 (1.7) | 4 | 80.0 (28.4–99.5) | 0.972 | 1.00 | |
| Indian | 99 (34.0) | 83 | 83.8 (75.1–90.5) | | 1.30 (0.14–12.37) | 0.821 |
| Nepalese | 187 (64.3) | 157 | 84.0 (77.9–88.9) | | 1.31 (0.14–12.12) | 0.813 |
| CMW community 4 | | | | | | |
| Sex | | | | | | |
| M | 460 (86.1) | 309 | 67.2 (62.7–71.5) | 0.001 | 1.00 | |
| F | 74 (13.9) | 21 | 28.4 (18.5–40.1) | | 0.19 (0.11–0.33) | <0.001 |
| Age, y | | | | | | |
| <29 | 257 (48.1) | 168 | 65.4 (59.2–71.2) | <0.001 | 1.00 | |
| 30–39 | 200 (37.5) | 108 | 54.0 (46.8–61.1) | | 0.62 (0.43–0.91) | 0.014 |
| 40–49 | 38 (7.1) | 17 | 44.7 (28.6–61.7) | | 0.43 (0.22–0.85) | 0.016 |
| ≥50 | 3 (0.6) | 2 | 66.7 (9.4–99.2) | | 1.06 (0.09–11.85) | 0.963 |
| Missing | 36 (6.7) | 35 | 97.2 (85.5–99.9) | | 18.54 (2.50–137.60) | 0.004 |
| Nationality | | | | | | |
| Other | 49 (9.2) | 11 | 22.4 (11.8–36.6) | <0.001 | 1.00 | |
| Indian | 62 (11.6) | 33 | 53.2 (40.1–66.0) | | 3.93 (1.70–9.07) | 0.001 |
| Nepalese | 155 (29.0) | 89 | 57.4 (49.2–65.3) | | 4.66 (2.22–9.79) | <0.001 |
| Bangladeshi | 268 (50.2) | 197 | 73.5 (67.8–78.7) | | 9.59 (4.65–19.77) | <0.001 |
| CMW community 5 | | | | | | |
| Sex | | | | | | |
| M | 437 (98.7) | 243 | 55.6 (50.8–60.3) | 0.007 | 1.00 | |
| F | 6 (1.4) | 0 | 0.0 (0.0–45.9) | | Omitted by model | NA |
| Age, y | | | | | | |
| ≤29 | 117 (26.4) | 67 | 57.3 (47.8–66.4) | 0.542 | 1.00 | |
| 30–39 | 193 (43.6) | 106 | 54.9 (47.6–62.1) | | 0.91 (0.57–1.44) | 0.687 |

| Characteristic | No. (%)† tested | SARS-CoV-2 seropositive No. | %‡ (95% CI) | p value | Univariable regression analysis OR (95% CI) | p value§ |
|---|---|---|---|---|---|---|
| 40–49 | 108 (24.4) | 54 | 50.0 (40.2–59.8) | | 0.75 (0.44–1.26) | 0.275 |
| ≥50 | 25 (5.6) | 16 | 64.0 (42.5–82.0) | | 1.33 (0.54–3.25) | 0.536 |
| Nationality | | | | | | |
| Other | 46 (10.4) | 17 | 37.0 (23.2–52.5) | <0.001 | 1.00 | |
| Indian | 160 (36.1) | 73 | 45.6 (37.7–53.7) | | 1.43 (0.73–2.81) | 0.297 |
| Sri Lankan | 63 (14.2) | 35 | 55.6 (42.5–68.1) | | 2.13 (0.98–4.64) | 0.056 |
| Nepalese | 174 (39.3) | 118 | 67.8 (60.3–74.7) | | 3.59 (1.82–7.08) | <0.001 |
| CMW community 6 | | | | | | |
| Sex | | | | | | |
| M | 1465 (97.3) | 939 | 64.1 (61.6–66.6) | <0.001 | 1.00 | |
| F | 40 (2.7) | 7 | 17.5 (7.3–32.8) | | 0.12 (0.05–0.27) | <0.001 |
| Age, y | | | | | | |
| ≤29 | 389 (25.8) | 258 | 66.3 (61.4–71.0) | 0.114 | 1.00 | |
| 30–39 | 644 (42.8) | 387 | 60.1 (56.2–63.9) | | 0.76 (0.59–0.99) | 0.045 |
| 40–49 | 374 (24.9) | 244 | 65.2 (60.2–70.1) | | 0.95 (0.71–1.29) | 0.753 |
| ≥50 | 98 (6.5) | 57 | 58.2 (47.8–68.1) | | 0.71 (0.45–1.11) | 0.132 |
| Nationality | | | | | | |
| Other | 173 (11.5) | 63 | 36.4 (29.2–44.1) | <0.001 | 1.00 | |
| Indian | 649 (43.1) | 408 | 62.9 (59.0–66.6) | | 2.96 (2.09–4.19) | <0.001 |
| Nepalese | 547 (36.3) | 366 | 66.9 (62.8–70.8) | | 3.53 (2.47–5.05) | <0.001 |
| Bangladeshi | 136 (9.0) | 109 | 80.1 (72.4–86.5) | | 7.05 (4.18–11.89) | <0.001 |
| CMW community 7 | | | | | | |
| Sex | | | | | | |
| M | 188 (100.0) | 122 | 64.9 (57.6–71.7) | NA | NA | |
| F | NA | NA | NA | | NA | NA |
| Age, y | | | | | | |
| ≤29 | 91 (48.4) | 61 | 67.0 (56.4–76.5) | 0.703 | 1.00 | |
| 30–39 | 54 (28.7) | 32 | 59.3 (45.0–72.4) | | 0.72 (0.36–1.44) | 0.346 |
| 40–49 | 29 (15.4) | 21 | 72.4 (52.8–87.3) | | 1.29 (0.51–3.25) | 0.588 |
| ≥50 | 11 (5.9) | 6 | 54.5 (23.4–83.3) | | 0.59 (0.17–2.09) | 0.414 |
| Missing | 3 (1.6) | 2 | 66.7 (9.4–99.2) | | 0.98 (0.09–11.28) | 0.989 |
| Nationality | | | | | | |
| Other | 24 (12.8) | 6 | 25.0 (9.8–46.7) | <0.001 | 1.00 | |
| Indian | 42 (22.3) | 2 | 4.8 (0.6–16.2) | | 0.15 (2.76–81.64) | 0.028 |
| Nepalese | 122 (64.9) | 114 | 93.4 (87.5–97.1) | | 42.75 (13.28–137.66) | <0.001 |
| CMW community 8 | | | | | | |
| Sex | | | | | | |
| M | 134 (96.4) | 92 | 68.7 (60.1–76.4) | 0.023 | 1.00 | |
| F | 5 (3.6) | 1 | 20.0 (0.5–71.6) | | 0.11 (0.01–1.05) | 0.056 |
| Age, y | | | | | | |
| ≤29 | 26 (18.7) | 16 | 61.5 (40.6–79.8) | 0.330 | 1.00 | |
| 30–39 | 70 (50.4) | 48 | 68.6 (56.4–79.1) | | 1.36 (0.53–3.48) | 0.517 |
| 40–49 | 33 (23.7) | 20 | 60.6 (42.1–77.1) | | 0.96 (0.33–2.76) | 0.942 |
| ≥50 | 10 (7.2) | 9 | 90.0 (55.5–99.7) | | 5.63 (0.62–51.37) | 0.126 |
| Nationality | | | | | | |
| Other | 26 (18.7) | 7 | 26.9 (11.6–47.8) | <0.001 | 1.00 | |
| Indian | 64 (46.0) | 46 | 71.9 (59.2–82.4) | | 6.94 (2.49–19.31) | <0.001 |
| Bangladeshi | 25 (18.0) | 18 | 72.0 (50.6–87.9) | | 6.98 (2.04–23.88) | 0.002 |
| Nepalese | 24 (17.3) | 22 | 91.7 (73.0–99.0) | | 29.86 (5.53–161.34) | <0.001 |
| CMW community 9 | | | | | | |
| Sex | | | | | | |
| Men | 164 (81.2) | 125 | 76.2 (69.0–82.5) | <0.001 | 1.00 | |
| Women | 38 (18.8) | 1 | 2.6 (0.1–13.8) | | 0.01 (0.00–0.06) | <0.001 |
| Age (years) | | | | | | |
| ≤29 | 30 (14.9) | 16 | 53.3 (34.3–71.7) | 0.201 | 1.00 | |
| 30–39 | 58 (28.7) | 33 | 56.9 (43.2–69.8) | | 1.16 (0.48–2.80) | 0.750 |
| 40–49 | 61 (30.2) | 38 | 62.3 (49.0–74.4) | | 1.45 (0.60–3.50) | 0.414 |
| 50+ | 53 (26.2) | 39 | 73.6 (59.7–84.7) | | 2.44 (0.95–6.25) | 0.064 |
| Nationality | | | | | | |
| All other nationalities | 28 (13.9) | 17 | 60.7 (40.6–78.5) | 0.766 | 1.00 | |
| Nepalese | 85 (42.1) | 51 | 60.0 (48.8–70.5) | | 0.97 (0.41–2.33) | 0.947 |
| Indian | 89 (44.1) | 58 | 65.2 (54.3–75.0) | | 1.21 (0.50–2.90) | 0.668 |
| CMW community 10 | | | | | | |
| Sex | | | | | | |
| M | 957 (100.0) | 620 | 64.8 (61.7–67.8) | NA | NA | |
| F | NA | NA | NA | | NA | NA |

| Characteristic | No. (%)† tested | SARS-CoV-2 seropositive | | | Univariable regression analysis | |
|---|---|---|---|---|---|---|
| | | No. | %‡ (95% CI) | p value | OR (95% CI) | p value§ |
| Age, y | | | | | | |
| ≤29 | 189 (19.7) | 116 | 61.4 (54.0–68.4) | 0.119 | 1.00 | |
| 30–39 | 392 (41.0) | 243 | 62.0 (57.0–66.8) | | 1.03 (0.72–1.47) | 0.887 |
| 40–49 | 273 (28.5) | 190 | 69.6 (63.8–75.0) | | 1.44 (0.98–2.13) | 0.067 |
| ≥50 | 103 (10.8) | 71 | 68.9 (59.1–77.7) | | 1.40 (0.84–2.32) | 0.199 |
| Nationality | | | | | | |
| Other | 69 (7.2) | 42 | 60.9 (48.4–72.4) | 0.061 | 1.00 | |
| Bangladeshi | 62 (6.5) | 37 | 59.7 (46.4–71.9) | | 0.95 (0.47–1.92) | 0.889 |
| Nepalese | 389 (40.6) | 238 | 61.2 (56.1–66.1) | | 1.01 (0.60–1.71) | 0.961 |
| Indian | 437 (45.7) | 303 | 69.3 (64.8–73.6) | | 1.45 (0.86–2.46) | 0.162 |

*CMW, craft and manual worker; NA, not applicable; OR, odd ratio; SARS-CoV-2, severe acute respiratory syndrome coronavirus 2.
†Percent positive of those tested.
‡Percent of total sample.
§Covariates with p value ≤0.05 in univariable analysis were considered as showing evidence for an association with SARS-CoV-2 seropositivity.

**Appendix Table 2.** Characteristics of CMWs and associations with SARS-CoV-2 real-time RT-PCR positivity, Qatar*

| Characteristic | No. (%)† tested | SARS-CoV-2 RT-PCR positive | | | Univariable regression analysis | | Multivariable regression analysis‡ | |
|---|---|---|---|---|---|---|---|---|
| | | No. | %§ (95% CI) | p value | OR (95% CI) | p value¶ | OR (95% CI) | p value# |
| Sex | | | | | | | | |
| M | 1,844 (91.5) | 100 | 5.4 (4.4–6.6) | 0.395 | 1.00 | | NA | |
| F | 172 (8.5) | 12 | 7.0 (3.7–11.9) | | 1.31 (0.70–2.43) | 0.396 | NA | NA |
| Age, y | | | | | | | | |
| <29 | 785 (38.9) | 39 | 5.0 (3.6–6.7) | 0.486 | 1.00 | | NA | |
| 30–39 | 787 (39.0) | 44 | 5.6 (4.1–7.4) | | 1.13 (0.73–1.76) | 0.581 | NA | NA |
| 40–49 | 351 (17.4) | 25 | 7.1 (4.7–10.3) | | 1.47 (0.87–2.46) | 0.148 | NA | NA |
| ≥50 | 93 (4.6) | 4 | 4.3 (1.2–10.6) | | 0.86 (0.30–2.46) | 0.778 | NA | NA |
| Nationality | | | | | | | | |
| Other** | 149 (7.4) | 15 | 10.1 (5.7–16.1) | 0.002 | 1.00 | | 1.00 | |
| Nepalese | 918 (45.5) | 46 | 5.0 (3.7–6.6) | | 0.47 (0.26–0.87) | 0.016 | 0.43 (0.22–0.82) | 0.011 |
| Bangladeshi | 265 (13.1) | 9 | 3.4 (1.6–6.3) | | 0.31 (0.13–0.74) | 0.008 | 0.44 (0.17–1.12) | 0.085 |
| Filipino | 112 (5.6) | 4 | 3.6 (1.0–8.9) | | 0.33 (0.11–1.03) | 0.055 | 0.49 (0.15–1.63) | 0.242 |
| Indian | 572 (28.4) | 38 | 6.6 (4.7–9.0) | | 0.64 (0.34–1.19) | 0.157 | 0.69 (0.33–1.45) | 0.329 |
| CMW community | | | | | | | | |
| 6†† | 832 (41.3) | 42 | 5.0 (3.7–6.8) | <0.001 | 1.00†† | | 1.00†† | |
| 1 | 92 (4.6) | 0 | 0.0 (0.0–3.9) | | Omitted by model | NA | Omitted by model | NA |
| 8 | 39 (1.9) | 0 | 0.0 (0.0–9.0) | | Omitted by model | NA | Omitted by model | NA |
| 4 | 363 (18) | 10 | 2.8 (1.3–5.0) | | 0.53 (0.26–1.07) | 0.078 | 0.63 (0.30–1.36) | 0.240 |
| 2 | 424 (21.0) | 32 | 7.5 (5.2–10.5) | | 1.54 (0.95–2.47) | 0.077 | 1.60 (0.91–2.79) | 0.099 |
| 3 | 266 (13.2) | 28 | 10.5 (7.1–14.9) | | 2.21 (1.34–3.65) | 0.002 | 2.44 (1.45–4.08) | 0.001 |

*CMW, craft and manual worker; Ct, cycle threshold; NA, not applicable; OR, odd ratio; RT-PCR, real-time reverse transcription PCR; SARS-CoV-2, severe acute respiratory syndrome coronavirus 2.
†Percent of total sample.
‡Pseudo-$R^2$ value in the multivariable logistic regression model is 3.4%.
§Percentage real-time RT-PCR positive of those tested.
¶Covariates with p value ≤0.2 in univariable analysis were included in multivariable analysis.
#Covariates with p-value ≤0.05 in multivariable analysis were considered predictors of real-time RT-PCR positivity.
**Includes all other nationalities that contributed <5% of the sample in each community. These are Egyptian, Ghanaian, Indonesian, Jordanian, Kenyan, Lebanese, Nigerian, Pakistani, Sri Lankan, Tunisian, and Ugandan.
††CMW community 6, which had the largest sample size, was chosen as a reference.

**Appendix Table 3.** Characteristics of 14 SARS-CoV-2 antibody-negative men who had a real-time RT-PCR positive result for SARS-CoV-2 at some point before this study was conducted, Qatar*

| Person no. | Age, y | Date in 2020 of real-time RT-PCR | Ct value | Date in 2020 of antibody test | Symptoms |
|---|---|---|---|---|---|
| 1 | 36 | May 13 | 35.5 | Aug 2 | Not recorded |
| 2 | 28 | May 4 | 34.1 | Aug 3 | Not recorded |
| 3 | 25 | May 4 | 35.1 | Aug 3 | Not recorded |
| 4 | 26 | Apr 28 | 22.0 | Aug 8 | Not recorded |
| 5 | 40 | Jun 6 | 35.5 | Jul 23 | Not recorded |
| 6 | 33 | Jun 9 | 23.7 | Jul 27 | Not recorded |
| 7 | 27 | May 12 | 35.8 | Jul 28 | Not recorded |
| 8 | 34 | May 13 | Unknown | Jul 28 | Not recorded |
| 9 | 31 | May 23 | 27.4 | Aug 20 | Not recorded |
| 10 | 31 | May 6 | 19.6 | Aug 20 | Not recorded |
| 11 | 33 | May 7 | 18.0 | Aug 20 | Not recorded |
| 12 | 39 | Jul 11 | 32.9 | Aug 20 | Not recorded |
| 13 | 44 | Aug 5 | 35.2 | Aug 20 | Asymptomatic |
| 14 | 34 | May 8 | 32.0 | Aug 24 | Not recorded |

*Ct, cycle threshold; RT-PCR, real-time reverse transcription PCR; SARS-CoV-2; severe acute respiratory syndrome coronavirus 2.

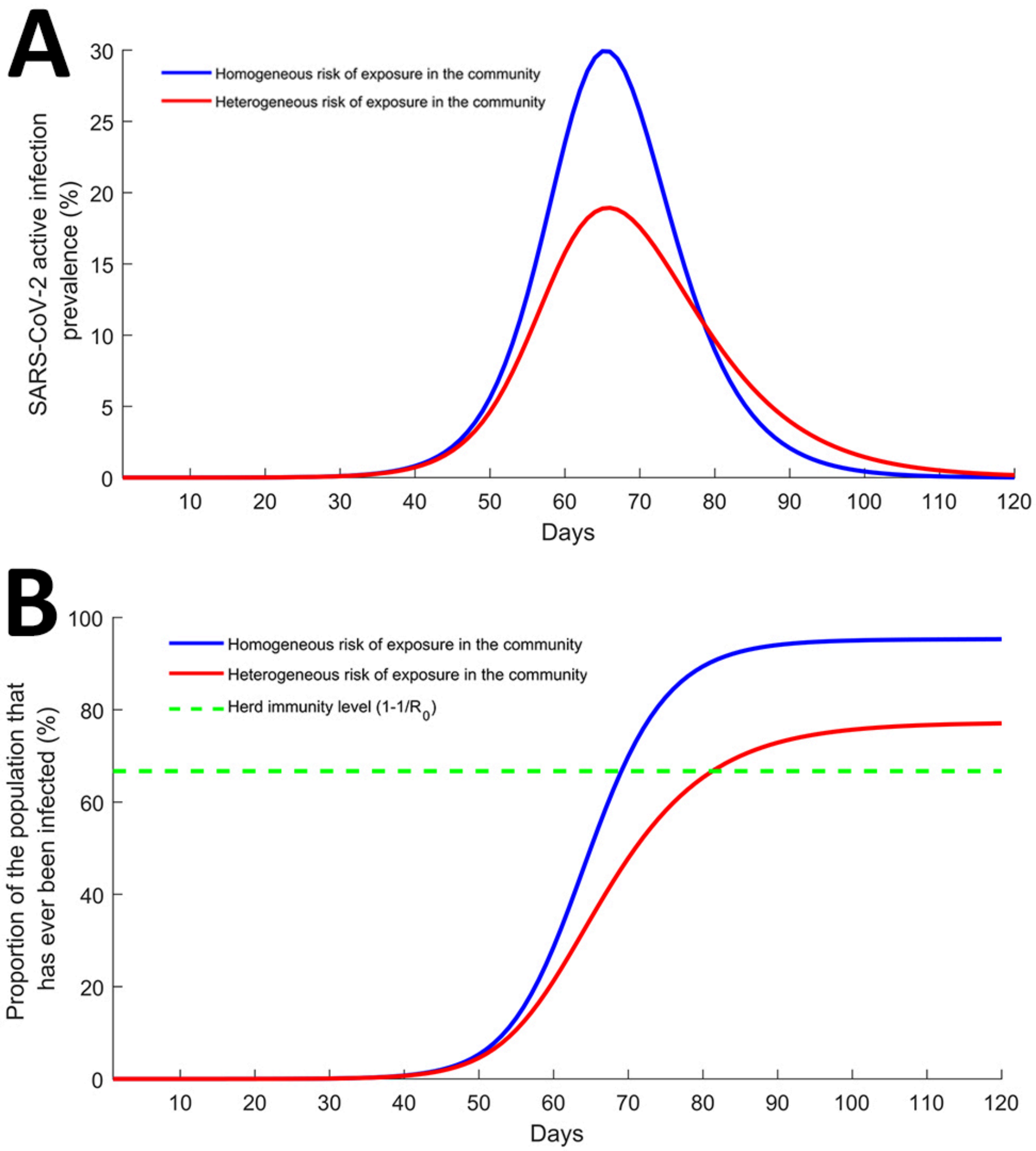


**Appendix Figure 1.** Herd immunity and heterogeneity in risk for exposure to the infection. SARS-CoV-2 active infection prevalence (A) and the proportion of the population that has ever been SARS-CoV-2 infected (B) in a community in which the risk for exposure is homogeneous versus in a community in which the risk for exposure is heterogeneous. In both of these scenarios, the basic reproduction number $R_0$ was assumed equal to 3 (*1,2*). These simulations were generated by using a classic age-structured susceptible-exposed-infectious-recovered mathematical model (*3*). Heterogeneity in the second modeled scenario was introduced through variable exposure risk by age. SARS-CoV-2, severe acute respiratory syndrome coronavirus 2.

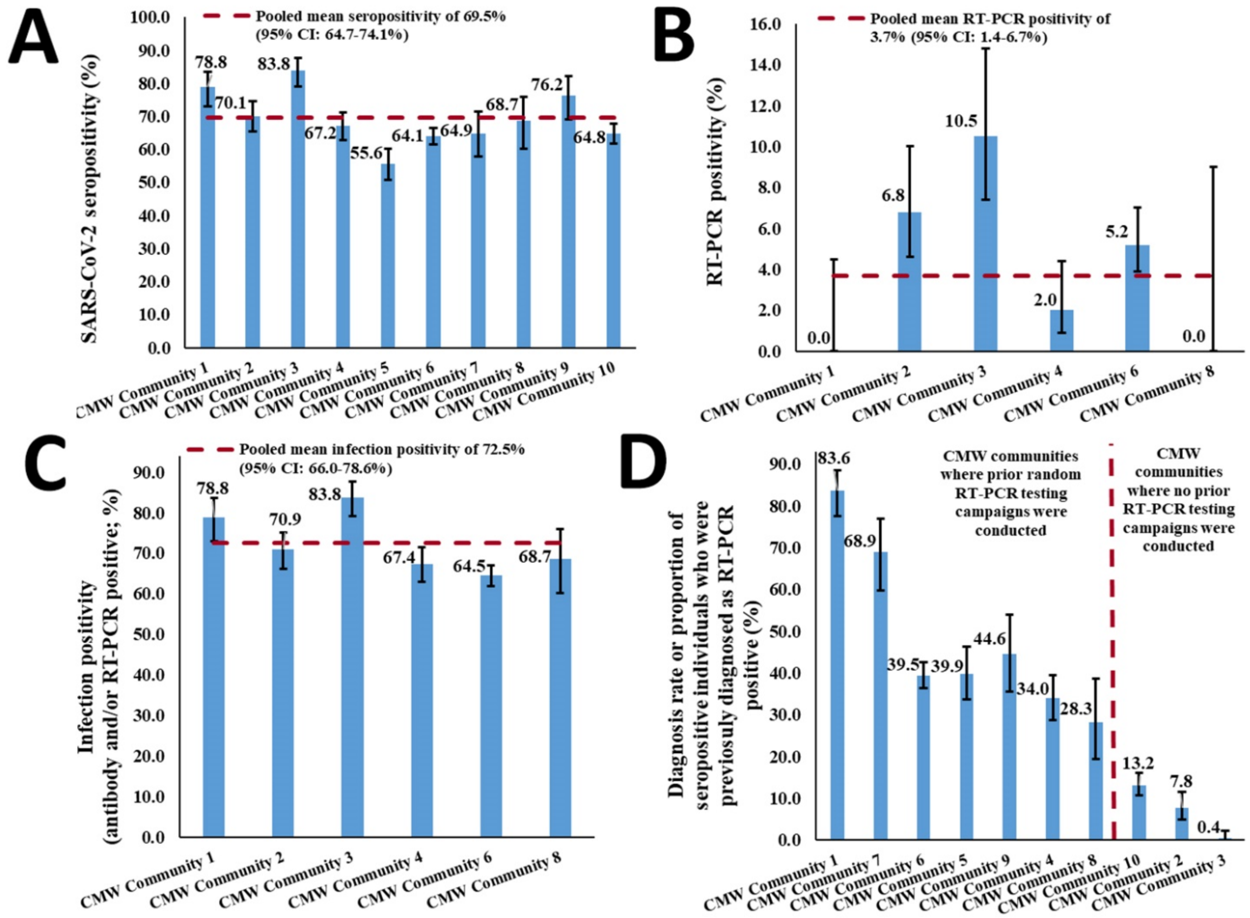


**Appendix Figure 2.** Measures of SARS-CoV-2 A) seropositivity (antibody positivity), B) real-time RT-PCR positivity, C) infection positivity (antibody or real-time RT-PCR positive), and D) diagnosis rate, among only male craft and manual workers (CMW) across the CMW communities. Panels B and D show results for only the 6 communities for whom real-time RT-PCR testing was performed. Error bars indicate 95% CIs. CMW, craft and manual workers; RT-PCR, real-time reverse transcription PCR; SARS-CoV-2, severe acute respiratory syndrome coronavirus 2.

## References

1. He W, Yi GY, Zhu Y. Estimation of the basic reproduction number, average incubation time, asymptomatic infection rate, and case fatality rate for COVID-19: meta-analysis and sensitivity analysis. J Med Virol. 2020;92:2543–50. PubMed https://doi.org/10.1002/jmv.26041

2. Online MIDAS. COVID-19 portal. COVID-19 parameter estimates: basic reproduction number [cited 2020 May 19]. https://github.com/midas-network/COVID-19/tree/master/parameter_estimates/2019_novel_coronavirus

3. Ayoub HH, Chemaitelly H, Seedat S, Makhoul M, Al Kanaani Z, Al Khal A, et al. Mathematical modeling of the SARS-CoV-2 epidemic in Qatar and its impact on the national response to COVID-19. J Glob Health. 2021;11:05005. https://doi.org/10.7189/jogh.11.05005
